# Deep Learning in Gait Parameter Prediction for OA and TKA Patients Wearing IMU Sensors

**DOI:** 10.3390/s20195553

**Published:** 2020-09-28

**Authors:** Mohsen Sharifi Renani, Casey A. Myers, Rohola Zandie, Mohammad H. Mahoor, Bradley S. Davidson, Chadd W. Clary

**Affiliations:** Center for Orthopaedic Biomechanics, University of Denver, Denver, CO 80208, USA; Casey.Myers@du.edu (C.A.M.); Rohola.Zandie@du.edu (R.Z.); Mohammad.Mahoor@du.edu (M.H.M.); Bradley.Davidson@du.edu (B.S.D.); Chadd.Clary@du.edu (C.W.C.)

**Keywords:** deep learning, convolutional neural network, gait analysis, total knee arthroplasty, wearable sensors

## Abstract

Quantitative assessments of patient movement quality in osteoarthritis (OA), specifically spatiotemporal gait parameters (STGPs), can provide in-depth insight into gait patterns, activity types, and changes in mobility after total knee arthroplasty (TKA). A study was conducted to benchmark the ability of multiple deep neural network (DNN) architectures to predict 12 STGPs from inertial measurement unit (IMU) data and to identify an optimal sensor combination, which has yet to be studied for OA and TKA subjects. DNNs were trained using movement data from 29 subjects, walking at slow, normal, and fast paces and evaluated with cross-fold validation over the subjects. Optimal sensor locations were determined by comparing prediction accuracy with 15 IMU configurations (pelvis, thigh, shank, and feet). Percent error across the 12 STGPs ranged from 2.1% (stride time) to 73.7% (toe-out angle) and overall was more accurate in temporal parameters than spatial parameters. The most and least accurate sensor combinations were feet-thighs and singular pelvis, respectively. DNNs showed promising results in predicting STGPs for OA and TKA subjects based on signals from IMU sensors and overcomes the dependency on sensor locations that can hinder the design of patient monitoring systems for clinical application.

## 1. Introduction

Quantitative assessments of movement quality in osteoarthritic (OA) and joint reconstruction patients, specifically spatial-temporal gait parameters (STGPs), provide valuable insight into gait patterns, activity type [1], risk of falling, and disease progression [2,3]. This diagnostic information is used in a number of applications that include development of personalized treatment plans, optimized post-operative rehabilitation, monitoring changes in mobility of patients after surgery [4,5,6,7], advancement of promising new interventions, and reducing overall medical costs [2]. Conventional methods for measuring gait characteristics that include motion capture (MOCAP) systems and force plates require a laboratory environment and expensive, time-consuming, equipment [8]. On the contrary, wearable sensors, specifically inertial measurement units (IMUs), are lightweight, inexpensive, and mobile. IMU’s measurement fidelity has improved significantly in recent years and have been used in various applications including 3D character animation, robotics, automotive vehicles, drones, and human motion measurement [9].

Processing streams of IMU data to extract clinically meaningful movement characteristics, such as activity classification, spatial-temporal parameters, gait pathology, and gait phase detection is challenging [10]. Several studies calculate spatial-temporal gait parameters by reconstruction of foot trajectories through double integration of the linear accelerations measured by IMUs. Sensor fusion techniques [11,12,13,14] and extended Kalman filters [15,16,17,18] are commonly used to reduce noise and improve measurement accuracy. These methods rely on identification of the zero-velocity condition of the foot during stance for gait segmentation. However, clear zero-velocity conditions are difficult to identify for patients with pathological gait or during highly dynamic activities like free running [19].

Data-driven approaches like deep learning have shown promising results in extracting complex patterns from data in the fields of computer vision, speech-recognition, and sequence modeling. Researchers have used deep learning on IMU-based movement data to classify different activities or quantify activity-specific movements [20,21,22,23]. Hannink et al. demonstrated the ability of deep-learning algorithms to recognize the non-linear relationships between raw IMU data and stride length as well as other STGPs [24]. Using a deep convolutional neural network trained on over 1220 strides from 101 geriatric patients, the algorithm predicted stride length with a mean error of −0.15 cm, which was considerably more accurate than previous integration-based methods [12]. Moreover, stride length predictions were robust to different methods of stride segmentation, improving the clinical applicability for patients with pathologic gait [25]. Similar results have been demonstrated using neural networks for measuring stride length during free running [17], but variability in foot strike patterns (e.g., heel strike versus toe strike) reduced accuracy highlighting the importance of population-specific datasets for best results.

Single body segment mounted IMUs (e.g., wrist or pelvis) are limited in calculation of certain STGPs such as number of steps, step cadence, or step distance which may not be adequate for clinical applications [26,27]. Incorporating IMUs on additional body segments (e.g., foot, shank, thigh, pelvis, or trunk) may provide access to additional gait metrics and improve gait characteristic predictions, especially for patient populations with pathologic movement characteristics [26,28,29,30]. Carcreff et al. demonstrated that IMUs placed on the shank and thigh yielded more accurate predictions of stride time, length, and velocity compared to feet mounted IMUs for children with cerebral palsy, particularly for those patients with increased disability [29]. Patients with progressive OA typically exhibit gait adaptations including decreased joint flexibility, increased stance time on the affected side, cadence, and double support time, and an overall increase in variability of spatial temporal parameters [31,32,33,34]. It is unclear how these gait adaptations progress over time and impact the prediction of gait mechanics using inertial sensors. Additionally, systematic studies quantifying optimal sensor combinations for the best performance across various patient populations are important to this field, but are lacking.

Thus, the purpose of this study was two-fold: (1) to access the ability of multiple contemporary deep neural network architectures to predict STGPs from IMU data in the OA and joint-replacement patient populations and (2) to determine the optimal sensor combination to maximize prediction accuracy. The results of this study will help patients suffering from OA who may go on to receive a total joint replacement benefit from the accurate real-time patient monitoring of STGPs to inform their treatment, surgical planning, and rehabilitation.

## 2. Materials and Methods

### 2.1. Gait Measurements of Osteoarthritic and Total Knee-Replacement Subjects

**Figure 1:**
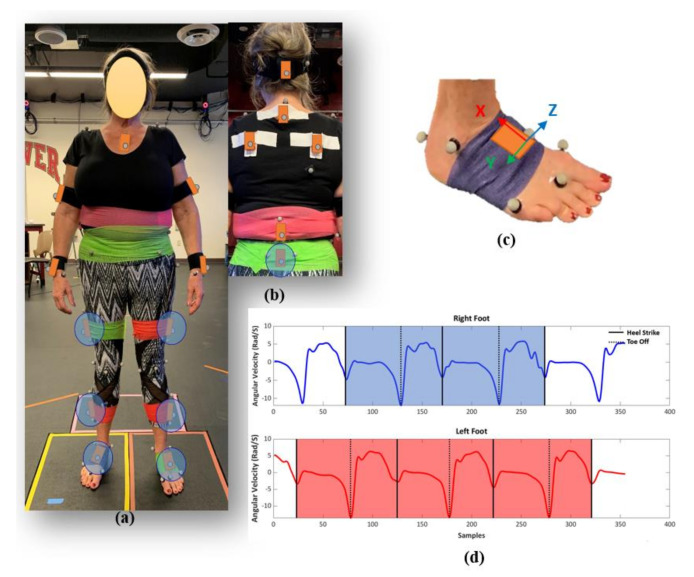
Subject suited up with markers and inertial measurement units (IMUs) (**a**) front, and (**b**) back view. IMUs circled in blue (feet, shanks, thighs, pelvis) were used in the supervised machine learning models. (**c**) IMU sensor attached on right foot with coordinate system, (**d**) a sample of segmented IMU data based on angular velocities of feet sensors.

Twenty-nine subjects, including 14 subjects with OA (Age = 67 ± 7, weight = 79 ± 12 kg, height = 168 ± 16 cm, 4 females and 10 males), 15 subjects with total knee arthroplasty (TKA) (Age = 68 ± 4, weight = 76 ± 14 kg, height = 164 ± 9 cm, 11 females and 4 males, 7 uni-lateral and 8 bi-lateral), participated in the study as part of a larger investigation. All participants signed a consent form prior to the experiment with IRB approval (# 1328728). Subjects were fitted with 71 reflective markers on anatomical landmarks and 17 IMUs on various limb segments and the trunk. For this study, only the 7 IMUs located on the feet, shanks, thighs [35,36], and pelvis [37] were used in the subsequent data analysis (Figure 1a,b). Subjects performed 15 trials of a 5-m walking task at three different speeds: self-selected, slow, and fast to cover the entire range of possible daily walking paces. During fast walking, subjects were instructed to walk at their maximum comfortable speed without running (brisk walking) typified by longer steps at a faster cadence. During slow walking, subjects were instructed to walk at their slowest speed, typified by shorter steps at a slower cadence. During the walking tests, synchronized data was collected from a 13 camera Vicon motion capture system (Centennial, CO), 4 Bertec force platforms (Columbus, OH), and IMUs (Xsens, Enschede, Netherlands) (Figure 1a). The sampling frequency of force data, MOCAP, and IMUs (free acceleration and angular velocity) were 1000 Hz, 100 Hz, and 40 Hz, respectively.

### 2.2. Gait Data Processing

MOCAP data were segmented into a full stride for each leg based on two successive heel strikes identified using the heel markers’ vertical position [38]. For each full stride, the heel strike and toe off times, spatial characteristics (step length, stride length, step width, and toe out angle), temporal characteristics (step time, stride time, stance time, swing time, single support time, and double support time), and general characteristics (cadence and speed) were calculated [39,40].

IMU data for each trial was up-sampled to 100 Hz and segmented into full strides for each leg based on the angular velocities of the feet sensors in the sagittal plane using the peak detection method (Figure 1c,d) [41,42]. The mean absolute error between heel strike and toe-off events identified using the IMU and MOCAP data were 0.02 ± 0.01 and 0.04 ± 0.01 s, respectively. Linear accelerations and angular velocities from the IMU-based coordinate systems for left legs were reflected about the medio-lateral axis to provide consistent anatomical directions for left and right limb segments. The IMUs’ six channels of acceleration and angular velocity data were normalized using the maximum sensor acceleration and angular velocity range. A zero-padding technique was used to ensure the IMUs’ data sets had a consistent length of 212 points prior to use in the deep-learning models [24]. The IMU data for each stride segment was labeled with the gait characteristics calculated using the MOCAP data for use in the subsequent supervised machine learning models. The gait data processing yielded 3778 segmented and labeled strides from the 29 subjects. A descriptive statistical analysis was conducted on measured spatial, temporal, and general gait parameters to characterize the dataset. This includes mean, standard deviation, coefficient of variation, and interquartile range for knee OA and TKA subject cohorts at three paces, slow, normal, and fast.

### 2.3. Preliminary Neural Network Architecture Benchmarking and Selection

Six contemporary multivariate time series neural network architectures were utilized to predict stride length from our subject cohort based solely on the feet IMU data (Table 1). Stride length and the feet IMUs were chosen to enable benchmarking prediction accuracy against published studies. For network training, 80% of strides from 26 of 29 subjects were randomly allocated to the training set and the remaining 20% of strides from the same subjects were allocated to a validation set. Strides from the final three subjects not included in the training set, one OA subject, one uni-lateral TKA subject, and one bilateral TKA subject, were allocated to a test set. Network prediction accuracy was assessed using 5-fold cross-validation, with training, validation, and test sets randomly reallocated for each fold of the cross-validation. Optimal architecture with the lowest errors for both validation and test sets was selected for conducting a design of experiment on prediction of STGPs with different sensor numbers and locations.

Neural networks were trained using a backpropagation and stochastic gradient descent optimization approach to minimize the loss function, mean square error (MSE), between the model-predicted and labeled stride length, using the form:

(1) $$\mathrm{MSE}=\frac{\sum_{i=1}^{n}{\left({\hat{y}}_{i}-y_{i}\right)}^{2}}{n}$$

where ${\hat{y}}_{i}$ was the model predicted stride length, y_i_ was the labeled stride length, and n was the total number of strides in the training set.

An adaptive learning rate optimization with a learning rate, beta-1, and beta-2 of 0.001, 0.9, and 0.999, respectively were used for training all networks [43] with a total epoch of 300. Once each network was trained, the predictive accuracy was quantified by calculating the mean error (ME) and the mean absolute error (MAE) between the predicted and measured stride lengths for both the validation and test sets. ME was calculated to enable comparison with previously published studies, whereas the MAE provides a better metric for true prediction accuracy. To enable an equitable comparison of the prediction accuracy across various gait characteristics with different magnitudes and units, the absolute error was divided by the mean of the labeled test data resulting in the normalized absolute percent error (NAPE).

### 2.4. Assessing Optimal Sensor Combinations for Each Gait Characteristic

Based on the result of the preliminary neural network architecture selection, the 1D convolution neural network (CNN) architecture proposed by Zrenner et al. was chosen for a larger design-of-experiment study on sensor combinations [19]. This network consisted of two convolutional layers followed by two max pooling layers, a flattening layer, and two fully-connected layers. Rectified linear unit (ReLu) activation functions were placed after each layer. Keras with a Tensorflow backend was used for training the architecture [44,45].

**Figure 2:**
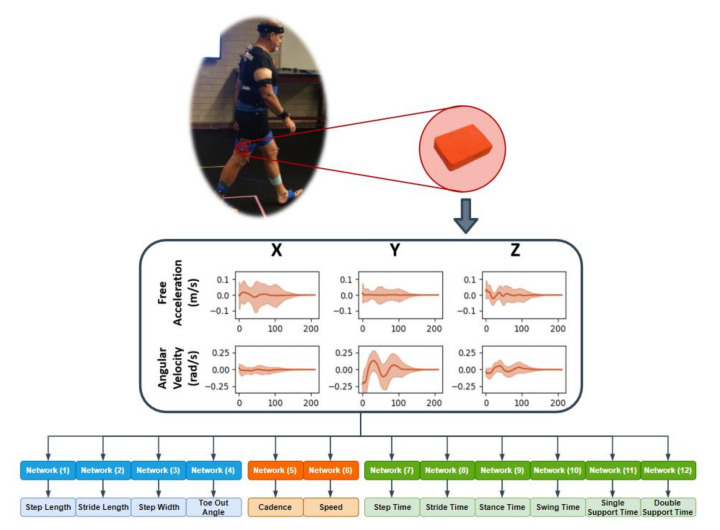
Workflow from in-vivo to spatial temporal gait parameter prediction.

A full factorial design of experiments was implemented to analyze the prediction accuracy based on 15 unique combinations of the feet, pelvis, shank, and thigh sensors (Table 2). Leveraging the ensemble approach proposed by Hannick et al., individual CNNs were trained using the segmented and labeled stride IMU data to predict each of the 12 spatial, temporal, and general gait parameters (Figure 2) for each unique sensor combination [24]. The same training set definitions, 5-fold cross-validation, and training approaches were used as in the preliminary analysis. Likewise, the same MAE and NAPE error estimations were calculated for each gait parameter with each sensor combination.

The Friedman test, which is a non-parametric statistical test analog to a repeated measures analysis of variance (ANOVA), was used to detect statistically significant differences in prediction accuracy (NAPE) across sensor combinations. Stepwise Dunn’s post hoc tests followed by Bonferroni correction due to multiple testing was performed to establish significant differences (new *p*-value: 0.05/105 = 0.000476). To determine an overall optimal sensor combination, sensor combinations were ranked based on Friedman ranking and averaged across all the gait parameters for each sensor combination [46].

## 3. Results

### 3.1. Spatial-Temporal Gait Parameters (STGPs) Statistical Analysis

The OA group demonstrated larger step width (+2.8 cm) and toe out angle (+4.9 deg), as well as smaller step length (−1.9 cm), stride length (−3.9 cm), double support time (−0.1 s), and speed (−3.4 cm/s) on average for three different paces compared to the TKA group. In general, OA patients demonstrated greater variation (standard deviation (SD), coefficient of variation (CV), and range) in all but two of the STGPs measured compared to TKA patients. Increases in variability (SD) was also observed for step length, stride length, cadence, and speed for fast trials in both OA and TKA groups compared to normal and slow paces (Table 3).

### 3.2. Benchmarking Neural Network Architecture

MAE for stride length ranged from 2.9 ± 2.6 cm to 6.9 ± 3.2 cm for the validation set and 7.6 ± 6.1 cm to 11.9 ± 7.1 cm for the test set (Table 4). The CNN architecture proposed by Zrenner et al. yielded the lowest MAE for both the validation and test data sets, and the lowest ME, NAPE, and ME standard deviation for the test set, indicating negligible bias and low variance in the stride length predictions. Additionally, this network architecture included only 148,529 parameters which was smaller than the other networks, reducing the computational cost of training the network and preventing overfitting.

### 3.3. Optimal Sensor Combinations for Gait Characteristics

**Figure 3:**
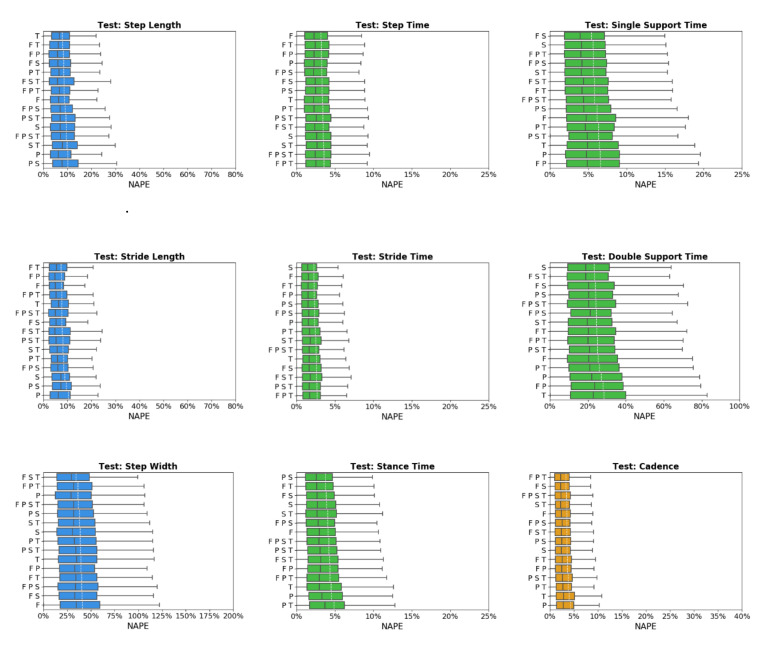

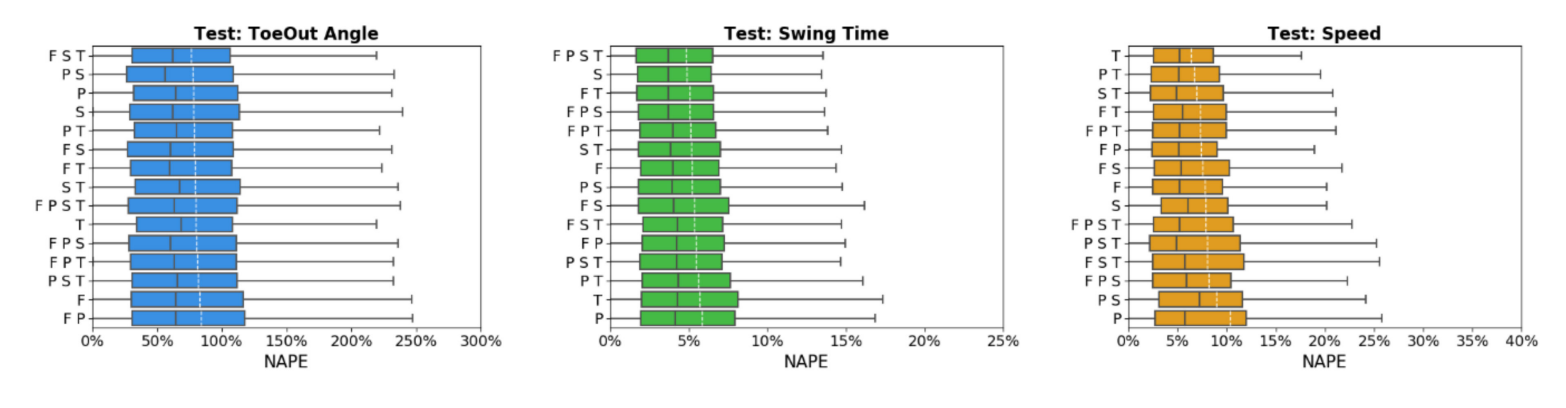
Normalized absolute percentage error (NAPE) of neural network for spatial (blue), temporal (green), and general (orange) gait parameters with various sensors configurations in the test set. Whiskers indicate 25% and 75% quartiles. For each gait parameter, sensor configurations are listed in order of increasing NAPE.

Across sensor combinations, network predictions for spatial gait characteristics were most accurate (lowest NAPEs) for step length (7.6 ± 6% − 9.7 ± 6.9%) and stride length (7.1 ± 5.7% − 9.6 ± 7.9%), followed by step width (34.9 ± 27.2% − 40.9 ± 32.9%) and toe-out angle (73.7 ± 50.9% − 80.6 ± 53.9%) (Figure 3). For temporal parameters, most accurate predictions were for step time (3.1 ± 2.9% − 3.5 ± 3.7%), stride time (2.1 ± 2.3% − 2.6 ± 3%), stance time (3.5 ± 3.5% − 4.8 ± 4.2%), and swing time (4.6 ± 4.1% − 5.6 ± 4.8%). Prediction errors increased for single support time (5.2 ± 4.4% − 6.6 ± 5.3%), and double support time (22.6 ± 18.1% − 28 ± 23.1%). For general parameters, cadence was predicted with the highest accuracy (3.2 ± 3.7% − 4.1 ± 4.6%) followed by speed (6.4 ± 5.2% − 9.6 ± 8.8%).

**Figure 4:**
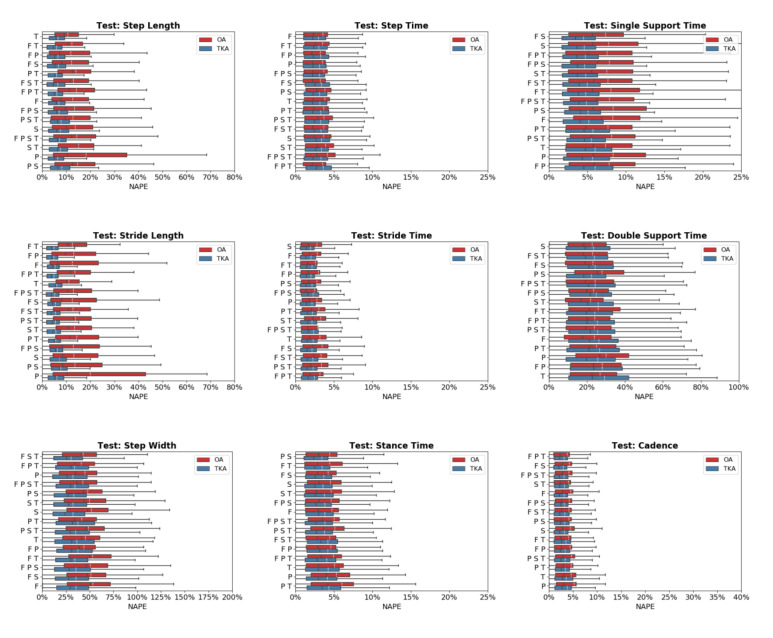

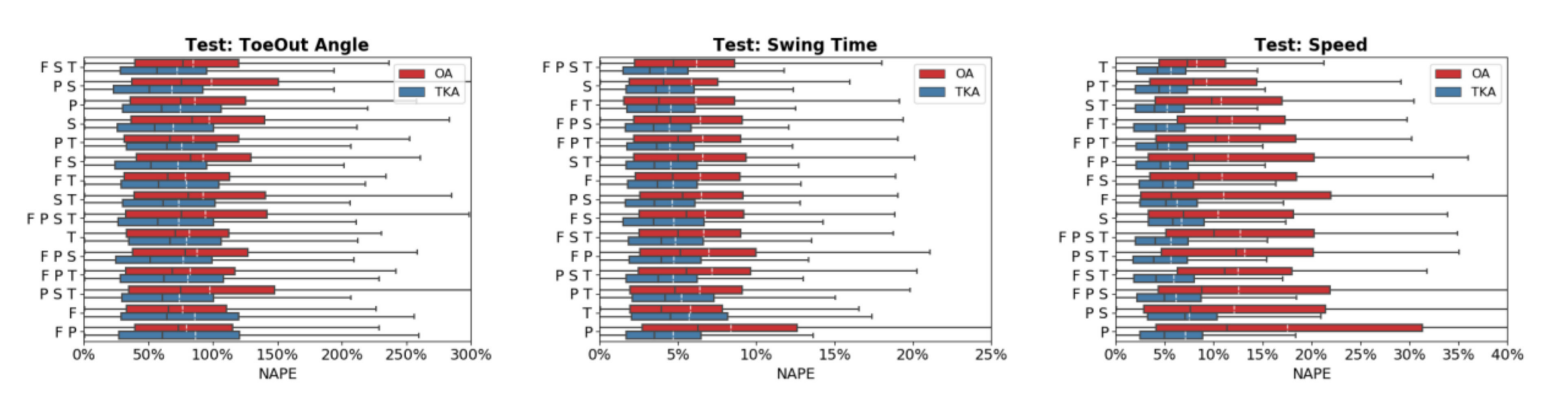
Normalized absolute percentage error (NAPE) of neural network predictions for all gait parameters and various sensors configurations grouped by subject cohort (OA and TKA) in the test set. For each gait parameter, sensor configurations are listed in order of increasing NAPE.

**Figure 5:**
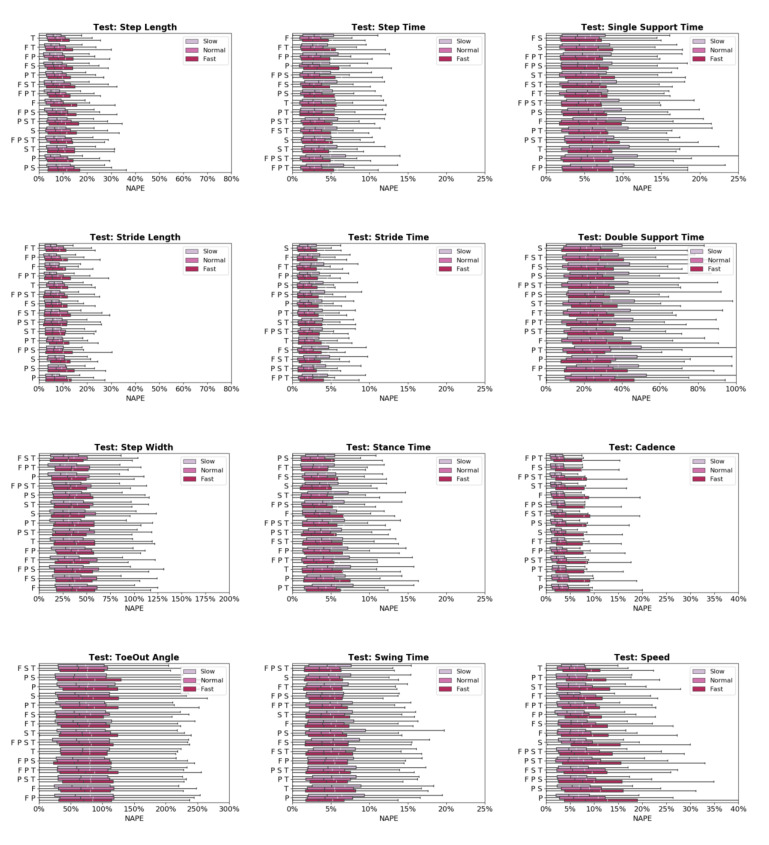
Normalized absolute percentage error (NAPE) of neural network predictions for all gait parameters and various sensors configurations grouped by gait pace (slow, self-selected, fast) in the test set. For each gait parameter, sensor configurations are listed in order of increasing NAPE.

Predictive accuracy was not equivalent between the OA and TKA cohorts, with generally larger prediction errors for the OA cohort (Figure 4). The OA cohort had larger mean (19.0%) and median (6.6%) NAPE across all sensor combinations and STGPs compared to TKA (mean NAPE = 14.7%, median NAPE = 4.6%). Fast walking also resulted in lower predictive accuracy relative to normal and slow walking. The mean and median NAPEs for fast walking were 17.7% and 6.22%, for normal walking were 15.8% and 4.8%, and for slow walking were 15.8% and 5.4% (Figure 5).

None of the sensor combinations consistently yielded the highest prediction accuracy for all variables. Sensor combinations were ranked based on NAPE for each gait parameter (Table 5). Overall, the feet-thigh (F T) configuration had the best average rank (5.1), followed by the feet-shank (F S, 6.2), and shank (S, 6.3) sensor combinations. The shank sensor combination consistently yielded the highest accuracy for temporal characteristics, ranking first or second for four of six temporal parameters. By contract, the pelvis (P) and pelvis-shank-thigh (P S T) sensor combinations consistently ranked among the least accurate with average ranks of 11 and 10.9, respectively.

The Friedman test indicated statistically significant differences (*p* = 0.001) between sensor combinations. Multiple pairwise comparisons based on Friedman ranking are displayed as homogenous subsets in Table 6. Similar to the mean NAPE ranking, feet-thigh and feet-shank sensor combinations ranked first and second in Friedman ranking. There was not a statistically significant difference between feet-thigh and feet-shank (adjusted *p*-value = 0.077) while there was a statistically significant difference between feet-thigh and the rest of the sensor combinations (adjusted *p*-value = 0.00). The pelvis sensor had the lowest accuracy with a significant difference compared to the other homogenous subsets of sensor combinations.

## 4. Discussion

The primary outcome of this study was the development of a robust deep-learning framework to predict diagnostic gait parameters for subjects with OA and TKA and investigate various sensor combinations on prediction accuracy. A simple ensemble deep neural network with two layers of 1D-CNNs demonstrated robust performance in predicting each STGP compared to more complex networks. A design of experiment conducted on 15 combinations of sensors and locations for different patient populations and gait paces revealed how the prediction accuracy of STGPs can change over different conditions and identification of an optimal sensor combination might be challenging. Overall, feet sensors combined with either shank or thigh sensors produced the highest accuracy for most STGPs and the isolated pelvis sensor showed the lowest accuracy.

The CNN architecture proposed by Zrenner et al. resulted in the lowest MAE and the lowest standard deviations for both the validation and test subject datasets with errors of 2.9 ± 2.6 cm and 7.6 ± 6.1 cm, respectively [19]. The CNN and ResNet model proposed by Hannik et al. and Wang et al. had the second and third lowest mean absolute errors of 8.2 ± 6.2 cm and 9.1 ± 6.4 cm [47]. Both Hannik et al. and Zrenner et al. published the mean and standard deviation of their models’ predictive error for stride length using unique datasets, enabling a direct comparison with our results [19,24]. Hannick et al. predicted stride length based on more than 1300 strides from 101 geriatric subjects, with a mean error of −0.15 ± 6.09 cm compared to our error of −2.2 ± 9.7 cm using the same network architecture. They used a larger number of subjects (n = 99) compared to our study (n = 29) which gives more unique data points for the network to train on. We induced additional variability in our dataset by asking subjects to walk at three different paces, all determined by the subjects. However, since this additional variability was mainly within-subject and may have a large amount of replication, it resulted in a slightly larger mean error compared to Hannink et al. In general, given large standard deviations in both studies, this difference was trivial. In this context, while our dataset had considerable variability, it likely had less variability than in the running dataset employed by Zrenner et al., which reported a mean predictive error in stride length of 2.5 ± 20.1 cm and a mean absolute error of 15.3 cm. The robustness of these CNN architectures for prediction of stride length point to the validity of using deep learning for this application, but also suggests that prediction accuracy is reduced when variability in the dataset is increased.

Direct comparisons of prediction accuracy between the current study and previous studies across all the STGPs are difficult due to differences in subject characteristics, dataset size, and experimental procedures. Comparable reported results from Hannick et al. for geriatric subjects, from Zrenner et al. for runners, and from Carcreff et al. for youths with cerebral palsy for sensor combinations and gait parameters are compiled in Table 7. Specifically, when comparing spatial parameter predictions using feet sensors, our results were within the range reported by previous studies. However, our results showed a larger mean error in prediction of stride length and step width compared to Hannink et al. that could be attributed to the larger number of subjects in Hannink et al. (n = 101) compared to our study. Diseases that induce pathologic movements, like OA, inherently increase the variability in gait parameters. The accuracy in prediction of the TKA group was higher than the OA group. The NAPE for OA was 19.0% and for TKA was 14.7%. When accounting for this limitation, our errors and standard deviations were comparable to previously reported results.

The neural networks trained on all sensor combinations predicted spatial, temporal, and general parameters with varying levels of accuracy. The NAPE averaged across all sensor combinations, for step length, stride length, step width, and toe-out angle were 8.6 ± 0.7, 7.8 ± 0.7, 38.5 ± 1.8, 77 ± 2%, respectively. The increased predictive error for step width and toe-out angle was likely associated with the smaller mean movements for those parameters, reducing the signal-to-noise ratio compared to the larger sagittal plane motions. For temporal parameters, the NAPE ranged from 2.3 ± 0.1% for stride time to 24.9 ± 1.5% for double support time. For the general parameters, the NAPE was 3.5 ± 0.2 and 7.5 ± 0.8% for cadence and speed, respectively. Descriptive statistical analysis on STGPs in our dataset revealed that neural network predictions were more accurate for the parameters with a lower coefficient of variation (CV). CV was defined as the ratio of the standard deviation to the mean which is an indicator of the dispersion of a probability distribution of data [48]. This was evident in the lower prediction accuracy observed for step width, toe-out angle, double support time, and speed with larger CVs compared to other parameters (Table 3).

The differences in predicted accuracy for OA versus TKA groups was multifactorial. First, there was more variability in the gait of OA subjects due to their pathology which makes it harder to predict certain STGPs. This higher variability for OA subjects was expressed by higher standard deviations and coefficients of variation for all gait parameters except toe-out angle, stride time, and cadence (Table 3). Second, the accuracy in prediction of STGPs was slightly higher at normal and slow walking compared to fast walking with mean NAPE of 15.8% for normal and slow, and 17.6% for fast walking. This aligns with findings by Zrenner et al. that indicated increasing speed could negatively impact predictive accuracy due to higher variability at fast walking (Table 3) [19]. Stressing the OA group with higher demand walking at a fast pace resulted in even greater variability and decreased predictive accuracy.

**Figure A1:**
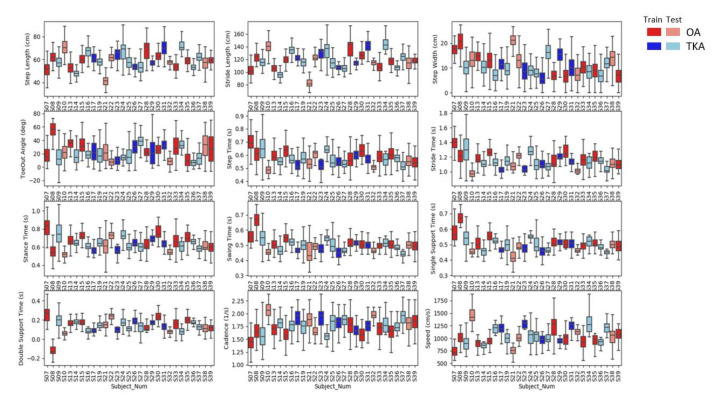
Boxplot associated with spatiotemporal gait parameters (STGPs) of each subject grouped by their knee status (OA and TKA) for training and test set.

**Figure A2:**
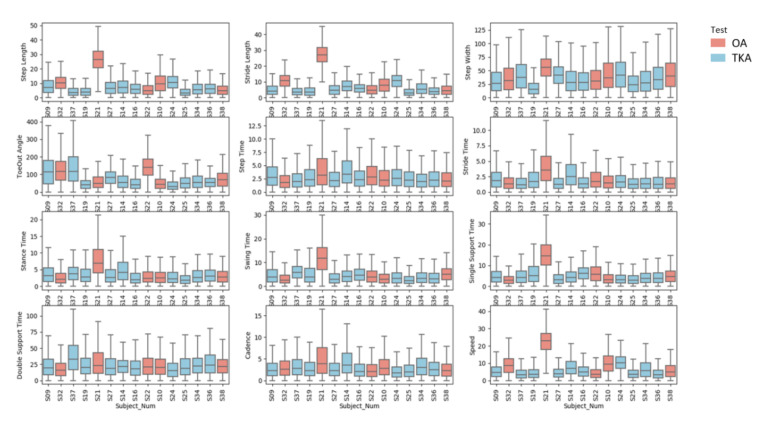
Boxplot associated with NAPEs of each test subjects grouped by their knee status (OA and TKA) for test set.

Perhaps most important, one of the randomly selected OA test subjects (subject S21, see Appendix A Figure A1 and Figure A2), walked with the shortest step length, shortest stride length, largest step width, and slowest speed among all subjects in the study, making this subject an outlier. Since our sample size was small, the impact of a single outlier was amplified and negatively affected prediction results. Jensen–Shannon divergence, which measures the similarity between two probability distributions, showed a larger divergence for subject S21 compared to the other two subjects in the same fold (S19 and S27). The divergences of step length, stride length, and step width for subject S21 were 5.67, 7.01, and 5.96 while for subject S19 and S27 the divergences were 0.18, 0.17, 0.10 and 0.38, 0.59, 0.67, respectively (see Appendix A Table A1). The divergence of S21 from the distribution of subjects used to train the CNNs resulted in poor performance, driving up the reported error for the OA cohort. Removing subject S21 from the test set reduced the mean and median NAPE from 19.0% and 6.6% to 17.3% and 4.8% which is comparable to the TKA group. Subject S21 had severe knee OA of the right knee which caused pain during activities of daily living and manifested in a noticeable limp on the affected limb compared to the other subjects in the OA cohort. Investigation of NAPE from the validation set revealed almost equal performance on both knee groups with mean and median NAPE of 7.7% and 2.9% for OA and 7.4% and 2.8% for TKA. The impact of subject S21 in the test set is an example of how CNNs result in poor performance when faced with data that are outside the distribution of the training data, which is one of the main challenges in the use of machine-learning models for real world applications. Hence, gaining intuition on training data set completeness is important prior to interpreting prediction accuracy. Out-of-distribution detection [49,50] has recently been recognized as an important area for developing trustworthy machine learning [49,50] and will be continually addressed in this work as patient numbers increase.

Our statistical analysis indicated statistically significant differences in accuracy between various sensor combinations tested across all conditions. The F-T combination was the highest-ranked sensor combination based on a Friedman test, showing a significant improvement in accuracy with respect to every other sensor combination except the F-S. It should be noted that although statistically significant, differences between the most and least accurate sensor combinations were small. The best sensor combination based on mean NAPE was F-T-S (15.25%) while the worst sensor combination was F-P (16.65%). Similarly, the Friedman test indicated the sensor combinations of F-T, F-S, and F-P-T were the top three ranked sensor combinations TKA subjects while T, F-S, and F-T were the top three ranked sensor combinations for OA subjects (see Appendix A Table A2a,b). The F-T and F-S were the common sensor combinations suitable for both OA and TKA groups. In addition, the F-T combination was also among the top three for slow, normal, and fast walking paces (see Appendix A Table A2c,e). As noted earlier, while the F-T sensor combination proved to be statistically better than other combinations, a 2–5% improvement in overall STGP prediction accuracy may be impactful during certain clinical applications. For instance, given the small difference in stride length at the normal pace between OA and TKA groups (~3 cm) higher accuracy predictions may be necessary for diagnostic purposes. However, higher accuracy may not be important for parameters with large differences between patient groups. This is an advantage of data driven modeling compared to other algorithm-based techniques in the prediction of STGPs. If the accuracy of STGPs is not largely impacted by sensor combination, there is freedom to design patient monitoring systems for specific patient groups based on other factors, such as cost and patient compliance. Feet sensors were necessary for stride segmentation during gait which is an input for the trained models. Therefore, including feet sensors is imperative for using a data-driven approach. Testing these sensor combinations on more complex tasks such as climbing stairs, sit-to-stand, and evaluating other joint kinematic and kinetic parameters would be necessary to clarify the value of using certain sensor combinations.

There are limitations to this study that should be considered. This study focused on gait to demonstrate the ability to predict STGPs from IMU data. In the OA population, other activities of daily living that place a greater demand on the subject will likely provide additional clinical value. The methods demonstrated in this study can be extended to predict analogous spatial temporal parameters for activities that include stair ascent/descent, sit-to-stand, and other high-demand activities. This study was also limited in the number of subjects that were included. This study demonstrated acceptable accuracy with 3778 segmented and labeled strides from the 29 subjects. Increasing the number of subjects and labeled strides will improve the predictive accuracy. Like other data-driven approaches, the trained network described in this study are only suitable for the selected population. There are also practical limitations to deploying our algorithm to a large patient population outside of a laboratory environment, including variability in sensor placement, reduced signal quality from low-cost IMUs, soft-tissue artifacts for high body mass index patients, and identification of patients with gait parameters outside the training data set. In order to implement this workflow for other populations with movement impairments that would benefit from patient monitoring, such as patients with cerebral palsy or stroke, the algorithm would need to be re-trained with inclusion of data from these populations. However, with this initial model architecture defined and the trained, a transfer-learning approach could be used on other populations to drastically reduce training time and the need for high volumes of data.

## 5. Conclusions

This study demonstrated that a deep-learning, data-driven approach was able to predict spatial temporal gait characteristics of OA and TKA patients based on signals from IMU sensors. Using a comprehensive analysis of various sensor combinations and their sensitivity to STGPs, patient population, and walking pace, our results showed that deep learning can overcome the dependency on sensor location that hinders the design of patient monitoring systems and negatively impacts patient compliance. Additionally, we demonstrated the importance sufficient variability in training and test data as a critical factor in the performance of DL models, especially for clinically relevant data with small sample sizes. A system that is able to leverage data streams from wearable sensors to produce real-time monitoring of STGPs in OA and TKA patients has the ability to improve clinical care and patient quality of life.

## Figures and Tables

**Table 1:** Contemporary multivariate time-series deep-learning models for prediction of stride length.

Reference	Models
Hannink 2017 [23]	Convolutional Neural Network (CNN)
Zrenner 2018 [17]	Convolutional Neural Network (CNN)
Wang 2017 [35]	Fully Convolutional Networks (FCN)
Wang 2017 [35]	Residual Network (ResNet)
Karim 2019 [36]	Multivariate Long Short-Term Memory Fully Convolutional Network (MLSTM-FCN)
Karim 2019 [36]	Multivariate Attention Long Short-Term Memory Fully Convolutional Network (MALSTM-FCN)

**Table 2:** Sensor combinations used in the design of experiment.

n	Feet	Pelvis	Shank	Thigh	Combinations
**1**	×				Feet (F)
**2**		×			Pelvis (P)
**3**	×	×			Feet Pelvis (F P)
**4**			×		Shank (S)
**5**	×		×		Feet Shank (F S)
**6**		×	×		Pelvis Shank (P S)
**7**	×	×	×		Feet Pelvis Shank (F P S)
**8**				×	Thigh (T)
**9**	×			×	Feet Thigh (F T)
**10**		×		×	Pelvis Thigh (P T)
**11**	×	×		×	Feet Pelvis Thigh (F P T)
**12**			×	×	Shank Thigh (S T)
**13**	×		×	×	Feet Shank Thigh (F S T)
**14**		×	×	×	Pelvis Shank Thigh (P S T)
**15**	×	×	×	×	Feet Pelvis Shank Thigh (F P S T)

**Table 3:** Descriptive statistic of spatial, temporal, and general parameters of dataset grouped by knee and pace (SD: Standard Deviation, CV: Coefficient of Variation, IQR: Interquartile Range).

Variable	Knee Status	Pace	Mean	SD	CV	Range	IQR
Step Length (cm)		Fast	66.6	10.4	15.6	50.0	9.0
	OA	Normal	56.7	9.1	16.1	54.6	9.2
		Slow	53.2	6.9	13.0	45.0	9.8
		Fast	66.0	9.9	15.1	46.7	10.8
	TKA	Normal	59.1	7.6	12.9	52.3	10.7
		Slow	53.7	6.5	12.1	37.1	8.4
Stride Length (cm)		Fast	132.9	20.3	15.3	91.5	16.3
	OA	Normal	113.0	17.4	15.4	88.4	17.0
		Slow	106.1	12.9	12.1	67.7	18.2
		Fast	132.1	18.9	14.3	93.4	17.4
	TKA	Normal	118.1	14.3	12.1	71.4	19.8
		Slow	107.2	12.3	11.5	67.2	14.5
Step Width (cm)		Fast	13.2	5.7	42.8	25.5	9.1
	OA	Normal	12.9	6.5	50.3	50.3	9.0
		Slow	12.4	5.1	41.3	24.5	7.5
		Fast	10.0	4.3	42.7	23.2	5.5
	TKA	Normal	10.0	4.9	49.3	27.2	6.5
		Slow	10.2	4.0	38.6	22.6	5.2
Toe out Angle (deg)		Fast	23.9	15.7	65.8	71.8	21.3
	OA	Normal	24.6	17.8	72.3	86.8	27.7
		Slow	27.4	16.7	60.9	72.4	26.1
		Fast	18.9	14.6	77.4	62.0	23.5
	TKA	Normal	20.8	15.9	76.5	105.0	23.4
		Slow	18.4	13.3	72.1	76.5	22.0
Step Time (s)		Fast	0.5	0.1	14.7	0.3	0.1
	OA	Normal	0.6	0.1	11.1	0.5	0.1
		Slow	0.7	0.1	13.6	0.6	0.1
		Fast	0.5	0.1	11.2	0.4	0.1
	TKA	Normal	0.6	0.1	9.9	0.4	0.1
		Slow	0.7	0.1	12.4	0.6	0.1
Stride Time (s)		Fast	0.9	0.1	13.7	0.6	0.2
	OA	Normal	1.1	0.1	10.0	0.7	0.2
		Slow	1.4	0.2	12.7	1.0	0.2
		Fast	1.0	0.1	10.2	0.6	0.1
	TKA	Normal	1.1	0.1	8.9	0.6	0.1
		Slow	1.4	0.2	11.7	1.0	0.2
Stance Time (s)		Fast	0.5	0.1	19.9	0.4	0.1
	OA	Normal	0.6	0.1	14.7	0.7	0.1
		Slow	0.8	0.1	16.7	0.8	0.2
		Fast	0.5	0.1	12.7	0.4	0.1
	TKA	Normal	0.6	0.1	11.2	0.5	0.1
		Slow	0.8	0.1	13.8	0.7	0.1
Swing Time (s)		Fast	0.4	0.1	11.7	0.3	0.1
	OA	Normal	0.5	0.1	11.4	0.4	0.1
		Slow	0.6	0.1	13.5	0.5	0.1
		Fast	0.4	0.0	9.8	0.3	0.1
	TKA	Normal	0.5	0.0	8.5	0.4	0.1
		Slow	0.6	0.1	11.9	0.5	0.1
Single Support Time (s)		Fast	0.4	0.1	13.6	0.3	0.1
	OA	Normal	0.5	0.1	12.2	0.4	0.1
		Slow	0.6	0.1	14.1	0.5	0.1
		Fast	0.4	0.0	9.7	0.2	0.1
	TKA	Normal	0.5	0.0	8.1	0.2	0.1
		Slow	0.6	0.1	11.8	0.5	0.1
Double Support Time (s)		Fast	0.0	0.1	270.0	0.5	0.1
	OA	Normal	0.1	0.1	60.3	0.5	0.1
		Slow	0.2	0.1	52.0	0.6	0.1
		Fast	0.1	0.0	70.3	0.3	0.1
	TKA	Normal	0.1	0.0	33.5	0.3	0.1
		Slow	0.3	0.1	30.0	0.6	0.1
Cadence (1/s)		Fast	2.2	0.3	15.3	1.6	0.5
	OA	Normal	1.8	0.2	11.0	1.8	0.3
		Slow	1.5	0.2	14.3	1.3	0.2
		Fast	2.1	0.3	12.6	1.9	0.2
	TKA	Normal	1.8	0.2	9.4	1.0	0.2
		Slow	1.4	0.2	12.8	1.2	0.2
Speed (cm/s)		Fast	146.7	23.0	15.7	93.6	30.8
	OA	Normal	99.8	20.2	20.2	114.8	21.5
		Slow	80.3	18.2	22.6	87.1	23.4
		Fast	139.7	26.1	18.7	122.0	38.5
	TKA	Normal	105.8	16.1	15.3	81.6	25.1
		Slow	77.5	14.2	18.3	74.7	17.3

**Table 4:** Stride length prediction errors, mean error (ME), mean absolute error (MAE), and normalized absolute percentage error (NAPE) for multiple contemporary network architectures.

Models	ME ± STD (cm) Validation Set	ME ± STD (cm) Test Set	MAE ± STD (cm) Validation Set	MAE ± STD (cm) Test Set	NAPE ± STD (%) Validation Set	NAPE ± STD (%) Test Set	Number Of Parameters
**CNN** [23]	0.5 ± 4.2	−2.2 ± 9.7	3.4 ± 2.9	8.2 ± 6.2	3 ± 2.5	7.2 ± 5.5	2,079,921
**CNN** [17]	**0.4 ± 3.7**	−2.4 ± 8.7	**2.9 ± 2.6**	**7.6 ± 6.1**	**2.5 ± 2.2**	**6.8 ± 5.5**	**148,529**
**FCN** [35]	−2.7 ± 3.9	−4.8 ± 9.1	8.4 ± 3.5	11.9 ± 7.1	7.3 ± 3	10.5 ± 6.3	277,121
**ResNet** [35]	0.5 ± 3.9	−1.9 ± 9.6	5.1 ± 3.2	9.1 ± 6.4	4.4 ± 2.8	8.1 ± 5.7	229,953
**MLSTM-FCN** [36]	1.0 ± 3.6	−1.2 ± 9.4	6.1 ± 3.1	9.5 ± 6.8	5.3 ± 2.7	8.3 ± 6	277,801
**MALSTM-FCN** [36]	1.0 ± 3.7	**−0.8 ± 9.0**	6.9 ± 3.2	10.3 ± 6.5	5.9 ± 2.7	9.1 ± 5.7	278,361
**Hannink et al.** [23]	NA	−0.15± 6.1	NA	NA			2,079,921
**Zrenner et al.** [17]	NA	2.5 ± 20.1	NA	15.3			

**Table 5:** Sensor combinations ranking based on mean NAPE.

	*F*	*P*	*F P*	*S*	*F S*	*P S*	*F P S*	*T*	*F T*	*P T*	*F P T*	*S T*	*F S T*	*P S T*	*F P S T*
**Step Length**	8	14	3	11	4	15	9	1	2	5	7	13	6	10	12
**Stride Length**	3	15	2	13	7	14	12	5	1	11	4	10	8	9	6
**Step Width**	15	3	11	7	14	5	13	10	12	8	2	6	1	9	4
**Toe Out Angle**	14	3	15	4	6	2	11	10	7	5	12	8	1	13	9
**Step Time**	1	4	3	12	6	7	5	8	2	9	15	13	11	10	14
**Stride Time**	2	7	4	1	12	5	6	11	3	8	15	9	13	14	10
**Stance Time**	7	14	11	4	3	1	6	13	2	15	12	5	10	9	8
**Swing Time**	7	15	11	2	9	8	4	14	3	13	5	6	10	12	1
**Single Support Time**	10	14	15	2	1	9	4	13	7	11	3	5	6	12	8
**Double Support Time**	11	13	14	1	3	4	6	15	8	12	9	7	2	10	5
**Cadence**	5	15	11	9	2	8	6	14	10	13	1	4	7	12	3
**Speed**	8	15	6	9	7	14	13	1	4	2	5	3	12	11	10
**Average Spatial**	10.0	8.8	7.8	8.8	7.8	9.0	11.3	6.5	5.5	7.3	6.3	9.3	4.0	10.3	7.8
**Average Temporal**	6.3	11.2	9.7	3.7	5.7	5.7	5.2	12.3	4.2	11.3	9.8	7.5	8.7	11.2	7.7
**Average General**	7.8	13.0	7.4	7.6	6.6	10.8	10.5	5.3	4.5	5.7	6.3	5.2	9.6	11.0	8.8
**Average**	7.6	11.0	8.8	6.3	6.2	7.7	7.9	9.6	5.1	9.3	7.5	7.4	7.3	10.9	7.5

Green is the highest rank. Red is the lowest rank.

**Table 6:** Homogeneous subsets based on Freidman ranking and asymptotic significances (0.05).

	Subset						
	1	2	3	4	5	6	7
**F T**	7.637						
**F S**	7.759	7.759					
**F P T**		7.792					
**F S T**		7.818	7.818				
**F P S T**			7.887	7.887			
**S**			7.911	7.911			
**F**				7.946	7.946		
**S T**				7.952	7.952		
**F P S**				7.987	7.987		
**F P**					8.036		
**P T**						8.181	
**T**						8.200	
**P S**						8.229	
**P S T**						8.280	
**P**							8.386
**Test Statistic**	6.519	2.018	3.228	5.417	9.667	3.830	.
**Sig. (2-sided test)**	0.011	0.365	0.199	0.247	0.022	0.280	.
**Adjusted Sig. (2-sided test)**	0.077	0.896	0.671	0.573	0.079	0.709	.

**Table 7:** Deep-learning accuracy comparison with previous studies for (**a**) spatial parameters, (**b**) general, and (**c**) temporal parameters.

**(a)**		**Spatial** **ME ± STD**			
		**Step Length** **(cm)**	**Stride Length** **(cm)**	**Step Width** **(cm)**	**Toe-Out** **Angle** **(deg)**
Feet	Our Results	−1.7 ± 5.2	−3.0 ± 8.7	1.1 ± 5.1	−3.2 ± 15.8
	Hannink	NA	−0.15 ± 6.09	−0.09 ± 4.22	NA
	Zrenner	NA	2.5 ± 20.1	NA	NA
	Carfcreff	NA	2.5 ± 3.7	NA	NA
Shank Thigh	Our Results	−0.6 ± 5.6	0.4 ± 9.7	0.85 ± 4.6	−3.7 ± 15.2
	Carfcreff	NA	7.5 ± 6.9	NA	NA
Average of all sensors	Our Test	−0.5 ± 0.6	−1.1 ± 1.3	0.6 ± 0.4	−3.5 ± 0.8
**(b)**		**General** **ME ± STD**			
		**Cadence** **(1/s)**		**Cadence** **(1/s)**	
Feet	Our Results	0.02 ± 0.1		0.02 ± 0.1	
	Hannink	NA		NA	
	Zrenner	NA		NA	
	Carfcreff	NA		NA	
Shank Thigh	Our Results	0.01 ± 0.09		0.01 ± 0.09	
	Carfcreff	NA		NA	
Average of all sensors	Our Results	0.04 ± 0.00		0.04 ± 0.00	
**(c)**		**Temporal** **ME ± STD**			
		**Stride Time** **(s)**		**Stance Time** **(s)**	**Swing Time** **(s)**
Feet	Our Results	–0.01 ± 0.04		–0.01 ± 0.03	0.01 ± 0.03
	Hannink	–0.00 ± 0.07		–0.00 ± 0.07	0.00 ± 0.05
	Carfcreff	0.00 ± 0.02		NA	NA
Shank Thigh	Our Results	0.00 ± 0.03		–0.01 ± 0.04	0.00 ± 0.03
	Carfcreff	0.00 ± 0.02		NA	NA
Average of all sensors	Our Results	0.00 ± 0.00		0.00 ± 0.00	0.00 ± 0.00

## Appendix A

**Table A1:** Jensen–Shannon (JS) divergence (relative entropy) between test subjects and training on fold with subjects S19, S21, and S27.

	JS(Training||S19)	JS(Training||S21)	JS(Training||S27)
**Step Length**	0.18	**4.67**	0.38
**Stride Length**	0.17	**7.01**	0.59
**Step Width**	0.10	**5.96**	0.67
**Toe Out Angle**	0.11	0.02	0.63
**Step Time**	0.01	0.26	0.80
**Stride Time**	0.02	0.31	1.26
**Stance Time**	0.01	0.14	0.63
**Swing Time**	0.02	0.33	0.55
**Single Support Time**	0.03	0.83	0.45
**Double Support Time**	0.08	0.04	0.29
**Cadence**	0.21	0.37	0.52
**Speed**	0.07	0.50	0.29

**Table A2:** Homogeneous subsets based on Freidman ranking and asymptotic significances (0.05) for (**a**) OA cohort, (**b**) TKA cohort, (**c**) slow pace, (**d**) normal pace, and (**e**) fast pace.

**(a)** **OA**	**Subsets**						
**Sensors**	**1**	**2**	**3**	**4**	**5**	**6**	
**T**	7.333						
**F S**		7.620					
**F T**		7.695	7.695				
**S**		7.776	7.776				
**F**		7.779	7.779				
**F P**			7.803				
**F P T**			7.809				
**F S T**			7.866				
**F P S**			7.985	7.985			
**F P S T**				8.115			
**P T**				8.144			
**S T**				8.164			
**P S**					8.445		
**P S T**					8.590		
**P**						8.876	
**Test Statistic**	.	9.049	9.052	9.927	6.536	.	
**Adjusted Sig. (2-sided test)**	.	0.103	0.330	0.070	0.077	.	
**(b)** **TKA**	**Subset**						
**Sensors**	**1**	**2**	**3**	**4**	**5**	**6**	**7**
**F T**	7.573						
**F S**	7.723	7.723					
**F P T**	7.807	7.807					
**F P S T**		7.822	7.822				
**F S T**		7.854	7.854				
**S T**		7.872	7.872				
**S**		7.878	7.878	7.878			
**F**		7.890	7.890	7.890			
**F P S**			8.029	8.029	8.029		
**P**				8.133	8.133	8.133	
**F P**					8.144	8.144	
**P S T**					8.197	8.197	
**P S**					8.294	8.294	
**P T**						8.297	
**T**							8.488
**Test Statistic**	5.659	5.695	8.917	9.540	8.562	8.379	9.928
**Adjusted Sig. (2-sided test)**	0.123	0.258	0.343	0.209	0.127	0.218	0.120
**(c)** **Slow**	**Subset**						
**Sensors**	**1**	**2**	**3**	**4**		**5**	
**F T**	7.361						
**F P T**		7.755					
**S**		7.774	7.774				
**F**		7.901	7.901				
**F S**		7.922	7.922				
**F P S T**		7.934	7.934				
**F P S**		7.939	7.939				
**F S T**		7.956	7.956				
**F P**		8.006	8.006				
**S T**		8.025	8.025				
**P**			8.074	8.074			
**P S**				8.294		8.294	
**P T**				8.304		8.304	
**T**						8.352	
**P S T**						8.403	
**Test Statistic**	.	16.124	12.904	6.791		4.860	
**Adjusted Sig. (2-sided test)**	.	0.067	0.184	0.157		0.530	
**(d)** **Normal**	**Subset**						
**Sensors**	**1**	**2**	**3**	**4**	**5**	**6**	
**F S**	7.726						
**F T**	7.737	7.737					
**F S T**	7.776	7.776					
**F P T**	7.778	7.778					
**F**		7.898	7.898				
**F P S T**			7.900	7.900			
**S T**			7.957	7.957			
**S**			7.976	7.976			
**F P S**			7.994	7.994			
**F P**				8.011			
**T**					8.133		
**P T**					8.143		
**P S**					8.215		
**P S T**					8.279		
**P**						8.476	
**Test Statistic**	3.552	9.311	4.664	10.546	3.401	.	
**Adjusted Sig. (2-sided test)**	0.757	0.092	0.690	0.093	0.782	.	
**(e)** **Fast**	**Subset**						
**Sensors**	**1**	**2**	**3**	**4**		**5**	
**F T**	7.503						
**F S**	7.712	7.712					
**F P S T**	7.762	7.762					
**S**	7.772	7.772					
**S T**		7.834					
**F S T**		7.846					
**F P T**		7.908	7.908				
**F P S**		8.011	8.011	8.011			
**P S T**			8.127	8.127		8.127	
**F P**			8.193	8.193		8.193	
**P T**				8.205		8.205	
**P S**				8.215		8.215	
**F**				8.238		8.238	
**T**				8.332		8.332	
**P**						8.342	
**Test Statistic**	8.994	12.448	10.675	13.328		10.304	
**Adjusted Sig. (2-sided test)**	0.106	0.110	0.050	0.080		0.226	
